# Assessment of Knowledge, Attitude, and Practice toward Antibiotic Use among Harar City and Its Surrounding Community, Eastern Ethiopia

**DOI:** 10.1155/2018/8492740

**Published:** 2018-08-08

**Authors:** ALemnesh Jifar, Yohanes Ayele

**Affiliations:** Department of Clinical Pharmacy, School of Pharmacy, College of Health and Medical Sciences, Haramaya University, Harar, Ethiopia

## Abstract

**Purpose:**

Community plays significant role in the process of emergence and spread of antibiotic resistance. The aim of this study is to assess knowledge, attitude, and practice toward antibiotic use among Harar city and its surrounding community, Eastern Ethiopia.

**Patient and Methods:**

A cross-sectional study was conducted among 384 subjects from February 1 to May 1, 2017, through interview using pretested structured questionnaires. The data was entered into EpiData 3.1 and analyzed using Statistical Package for Social Science for windows version 20.

**Results:**

A large number of the respondents (83%) replied that antibiotics speed up the recovery from coughs and colds. The majority of participants (78.4%) agreed that the unnecessarily use of antibiotics can increase the resistance of bacteria. Many respondents agreed on the importance of taking full dose (92.1%) and not to keep antibiotics for future use in their home (87.2%). They (90%) also had belief that antibiotics should not be shared from family or friends without a physician consultation and significant participants (73.1%) emphasized on the need for prescription to collect antibiotics from pharmacy. Around 79% of the subjects reported the use of antibiotic 1 year prior to study period at least once. During this period many subjects (65.3%) self-prescribed antibiotics without consulting physicians.

**Conclusion:**

In the present study, widespread use of antibiotics was reported, most of this antibiotics being accessed without prescription. Respondent exhibited poor knowledge and attitude toward antibiotics use. There were also malpractices such as failing to take full dose. Therefore, educational interventions on antibiotics use and its association with drug resistance are needed to promote judicious use of antibiotic. Introducing and enforcing antibiotics regulations should be also considered to reduce antibiotics self-prescription.

## 1. Introduction

Antibiotics have played monumental role in the infectious disease control and management since their discovery. Their use in both preventive and curable therapy have saved life of countless patients and improved patient care in general [1]. However, their effectiveness is seriously endangered by the emergence of resistant microorganisms [2]. Infections with resistant organisms have been associated with delayed duration of therapy, increased hospital stay, increased mortality, use of additional drugs, and laboratory tests and other resources increasing cost of treatment [3].

Complex factors derive the process of antimicrobial resistance development. Although evolutionary process combined with closely living world population plays significant role for emergence and spread of resistant microorganism, one would not underestimate the impact of extensive and unnecessary antimicrobial use [4, 5]. The inappropriate use of antibiotics could result from a complex interaction among various factors: prescriber behaviors and knowledge, diagnostic uncertainty, patient demand, the poor patient-prescriber interaction and macrolevel factors such as sociocultural, economic, and health care regulatory policy [5]. Furthermore, patients' knowledge, beliefs, and attitudes, their expectations, and experience with antibiotics have been contributing factor for spread and emergence of resistant microorganisms [6]. From patients perspective inappropriate antibiotics use such as failure to complete treatment, skipping of doses, reuse of leftover medicines, misuse of antibiotics in treating viral infections, and self-medication with antibiotics have been reported [2].

Self-medication to certain extent has a positive impact on individuals and health care systems in general if practiced correctly. However, studies conducted in different settings indicate high prevalence of self-medication practice ranging from 38.7% to 83% often associating it with irrational use of medications [7–10]. Increased self-medication practice could lead to wastage of resources, resistance of pathogens, and adverse reaction, incorrect self-diagnosis, delays in seeking appropriate care, and risk of dependence and drug abuse [11, 12]. The use of antibiotics without physicians recommendation is a most common contributing factor for antibiotic resistant [13, 14]. For instance, study conducted among university students in Karach reported 47.6% use of antibiotics without doctors' prescription six months prior to study [15]. Furthermore, about 40% of Chinese university students used antibiotics without prescription [16]. This kind of practice could lead to inappropriate use of antibiotics ultimately contributing toward emergence and spread of antibiotics resistance. Poor knowledge and attitudes toward self-medication with antibiotics have been attributed to increased use of antibiotics [16, 17].

In developing countries where infectious disease is rampant with little medical access and poor regulation, the problem is only expected to be worse. Moreover, community-based studies on knowledge and attitudes concerning antibiotics are few and those that have been conducted reported poor knowledge and attitudes [17]. In Harar city and its surrounding inhabitants, no such study has been done as far as our knowledge goes. Hence, it is very important to determine understanding and belief of communities toward antibiotics usage. Thus, the aim of this study is to explore the current knowledge and attitudes towards antibiotic usage among the general public of Harar city and its surrounding residents.

## 2. Materials and Methods

### 2.1. Study Area and Period

A facility-based cross-sectional study was conducted in Harar city, located 526 km East of Addis Ababa from February 1 to May 1, 2017. During the study period, there were 16 pharmacies and 44 drug stores in the Harar city.

### 2.2. Study Population

All clients who visited selected private drug retail outlets were included in the study. Clients were excluded if they were health care professionals. Sample size was determined by using single population formula considering the P value of 50% for antibiotics use during past one year, CI of 95% and marginal error of 5% giving total sample size of 384.

### 2.3. Sampling Technique

Stratified sampling technique was used to select representative drug retail outlet (from both pharmacies and drug stores). Four pharmacies and 10 drug stores were selected randomly. The total sample was distributed across each selected drug retail outlet based on the average clients flow. Study subjects were selected by using systematic random sampling technique using calculated K-value (total expected clients during study period divided by number of samples).

### 2.4. Data Collection and Analysis

The data was collected through exit client interview using structured questionnaires adapted from previous study [18] and customized to suit our purpose. The pilot study was conducted on five percent of total sample size to check the reliability and validity of the tools. The data was collected by two data collectors, graduating pharmacy students. Training was given to data collectors on the content of data collection tools and on the approach of interviews. The questionnaires used have four sections: sociodemographic characteristics such as age, sex, marital status, educational level, occupation, and monthly income; nine antibiotics use questionnaires were designed to assess the utilization practice of antibiotics among community; eight knowledge questionnaires were used to assess the participants knowledge; and seven attitude questionnaires were used to assess the participants attitude toward the antibiotics. The knowledge questionnaires had “Yes”, “No”, and ‘I do not know' options. The attitude questionnaires were designed according to the five-point Likert scale having options of “strongly disagree”, “disagree”, “neutral”, “agree”, and “strongly agree”. The antibiotics use questionnaires consisted of nine questions with different answering modality mainly “Yes” and “No”, closed ended questions with choices. The questionnaires consisted of the following questions; whether they had prescription for antibiotics in one year before the study, how many prescriptions they get for antibiotics, compliance to prescription, followed by reason for noncompliance. We also assessed self-medication practice with antibiotics and from where they did access the antibiotics. Finally, the sharing practices of the antibiotics were also assessed. The data was entered into EpiData 3.1 and analyzed using Statistical Package for Social Science for windows version 20. Descriptive statistics, frequencies, and percentage were used to summarize the result and presented using tables.

### 2.5. Ethical Consideration

The research protocol was reviewed and approved by the school delegate of Institutional Health Research Ethics Review Committee, Haramaya University. A formal permission letter was obtained from College of Health and Medical Sciences and submitted to drug retail outlets managers and they were also communicated the purpose of the research and how their premise is selected. Before the interview, verbal consent was obtained from each participant. The issue of assuring privacy and confidentiality was given more attention during the study by keeping the patient's name anonymously and using identification number to refer each study participants.

## 3. Results

### 3.1. Sociodemographic Characteristics of Participants

As it can be seen from Table 1, more than half of the participants (52.1%) were females and 184 (47.9%) were males. A majority of the participants 211(68.49%) were aged 18-34 years and only 129(33.6%) had higher education. Significant number of the participants had monthly income of less than 1200 ETB.

### 3.2. Knowledge of Antibiotics Use among Participants

As it is shown on Table 2, many of respondents agreed on the need for different antibiotics to cure different diseases (96%). However, participants had different views about whether antibiotics were effective against coughs and colds, viruses, and bacteria. A large number of the respondents (83%) replied that antibiotics speed up the recovery from most coughs and colds. Many respondent agreed that antibiotics are effective against bacteria (90%) and about just less than half (47%) claimed that antibiotics are effective against viruses. Encouragingly, the study population was more knowledgeable about antibiotic resistance. The majority of participants (78.4%) agreed that the unnecessarily use of antibiotics can increase the resistance of bacteria (Table 2).

### 3.3. Attitude towards Antibiotics Use among Participants

As it is depicted in Table 3, the majority of the respondents agreed on the necessity of completing the antibiotic course (92.1%) and not to keep antibiotics for future use (87.2%).They (90%) also had belief that antibiotics should not be obtained from family or friends without a physician consultation and a large portion of participants (73.1%) emphasized on the need for prescription to purchase antibiotics from pharmacy. However, substantial respondent had poor attitude toward the use of antibiotics for treatment of sore throat and cough, 55.7% and 63.77%, respectively.

### 3.4. Antibiotics Use among Participants

Overall, 304 (79.2%) of the responders reported the use of antibiotic 1 year prior to the study period. From those who used antibiotics, more than half (53.5%) of the participants used the antibiotics once, 39% used twice, and 7.5% used them three times. During this period, many of them, 111 (65.3%), self-prescribed antibiotics without consulting physicians. A large number of the participants (91.2%) got the antibiotics from pharmacy, whereas a few shared them with family and friends, 5.3% and 3.5%, respectively. When asked if they did take their last antibiotic for complete course, only 202(66.4%) completed the regimen. The main reason reported was feeling better 69(67.6%), followed by forgetting 20(19.6%), and developing side effects 13(12.7%).

## 4. Discussion

The aim of this study was to assess knowledge, attitude, and practice of the community with regard to antibiotics use. In the present study, widespread use of antibiotics was reported, most of it being accessed without prescription. Respondent exhibited poor knowledge and attitude toward antibiotics use. There were also malpractices such as failing to take full dose, purchasing antibiotics without prescription.

Although a large number of participants (96%) agreed that different antibiotics are needed to treat different disease, a majority of them (83%) had misunderstanding that antibiotics can be used to speed up recovery from cough. This finding is different from study conducted in Kuwait and Saudi Arabia in which they reported 54.4% [18] and 52.2% [19]. The difference seen could be due to sociodemographic and setting difference. Given the widespread occurrence of upper respiratory illness, such understanding can lead to inappropriately increased use of antibiotics adding to antimicrobial resistance crises.

In this study, many participants (78.3%) agreed that unnecessary use of antibiotics could lead to antimicrobial resistance. This finding is slightly higher than study done in Bahir Dar (69.7%) [20], Jordan (50%) [21], and Namibia (72%) [22]. Although this is encouraging finding, only nearly one-third (31%) of participants appreciated the problem as worldwide. This result is consistent with report from Jordan [21] and it highlights yet another knowledge gap among the users.

The present study revealed positive attitude on the necessity of taking full course of the antibiotics regimen (92.1%) and not using the leftover medicine (87.2%). This finding is in line with other study in which only 17% respondent kept antibiotics in their home for future use [23]. However, it is far better than Namibian study in which 28.5% of users kept antibiotics in their home for future use [24].

Regarding appropriate ways of getting antibiotics, participants displayed encouraging attitude. In present study, most respondent agreed on the need for physician consultation before purchasing antibiotics (90%) and getting prescription to purchase antibiotics (73.1%). This finding is just higher than study done in Saudi Arabia which they reported, 76.6% and 66.6%, respectively [18].

In current study, about 79% clients reported the use of antibiotics at least once in the 12 months prior to study. This report is comparable to study done in Namibia (80%) [22]. However, it is quite more than study done in Lithuania 24.9% [24] and in Bahir Dar 35.9% [25]. Many factors could be attributed to the difference seen including the pattern of disease prevalence in the given area and data collection period. For example, upper respiratory illness which clients tend to use antibiotics often shows seasonal variation. On the other hand, one could argue the effect of accessibility of antibiotics since easy access might encourage clients to use antibiotics for minor illness.

The current study indicates about 65% of antibiotics use without prescription. This outcome is interesting as most of the participants believed in the need for prescription to get antibiotics. This disparity may indicate roles of other factors than knowledge and belief of the users when it comes to practice. In addition, significant variation was observed among literatures regarding the use of antibiotics without prescription. For instance, about 76% of antibiotics use without prescription was reported in India [26], 32.7% in Italy [27], 28.8% in Saudi Arabia [19], and 9% in Hong Kong [28]. This difference might be because of variation of regulation from one area to other and its enforcement.

As to the sources of antibiotics, the drug outlets were the main sources (91.2%). In this study, a majority of the participants reported the use of antibiotics without prescription. This finding implies extensive practice of dispensing antibiotics without prescription paper. Hence, it is very important to look at the problem from pharmacy professionals' perspective and take necessary measures including introducing more stringent regulation on dispensing without prescription. In contrast to other studies [21, 26], in the current study, sharing of antibiotics with family and friends was minimal.

In our study, completing the course of the regimen was another area where the participants demonstrated deficiency. In this study, about 34% of the participants failed to complete the antibiotics course. Encouragingly, this result is less than study done in China, 49.8% [29], and Malaysia, 55.9% [30]. However, it is significantly greater than Namibian in which they reported only 20% of discontinuation of the dose, respectively [22].This inappropriate practice has been reported in other studies and has been associated with increased antimicrobial resistance [4, 5].

## 5. Conclusion

In the present study, widespread use of antibiotics was reported, most of this antibiotics being accessed without prescription. Respondent displayed poor knowledge particularly in regard to the role of antibiotics in minor viral illness. Participants had poor attitude toward the use of antibiotics for cold and sore throat. There were also significant malpractices such as failing to take full dose, purchasing antibiotics without prescription. Hence, educational interventions on antibiotics use and its association with drug resistance are needed to promote judicious use of antibiotic. Enforcing antibiotic regulations at a national level is also a key measure to reduce over the counter sales which in turn reduce antibiotics self-prescription.

## Figures and Tables

**Table 1:** Sociodemographic characteristics of participant in Harar city and its surrounding residents from February 1 to May 1, 2017 (n=384).

**Socio-demographic characteristics**	**Number (**%**)**
**Age**	
18-34	263(68.5)
35-64	88(22.9)
≥65	33(8.6)
**Sex**	
Male	184(47.9)
Female	200(52.1)
**Residence **	
Urban	283(73.7)
Rural	101(26.3)
**Marital status**	
Married	264(68.7)
Single	120(31.3)
**Education **	
No formal education	51(13.3)
Primary education	111(28.9)
High school	93(24.2)
College diploma and above	129(33.6)
**Occupation**	
Merchant	92(24.0)
Government employ	83(21.6)
Student	54(14.0)
Housewife	70(18.3)
Farmer	57(14.8)
Daily laborer	22(5.7)
Other	6(1.7)
**Monthly income in ETB** **∗**	
≤1200	167(43.5)
1201-2500	76(19.8)
>2500	141(36.7)

*∗*ETB, Ethiopian Birr, $1=28ETB.

**Table 2:** Knowledge towards use of antibiotics among Harar city and its surrounding residents, February 1 to May 1, 2017 (n = 384).

**Knowledge Statement **	**Yes**	**NO**	**I don't know**
Different antibiotics are needed to cure different disease	369(96%)	6(1%)	9(2%)

Antibiotics are effective against bacteria	346(90.1%)	13(3.4%)	25(6.5%)

Antibiotics speed up the recovery from most coughs and colds	319(83.1%)	51(13.3%)	14(3.6%)

Antibiotics are effective against viral diseases	179(46.6%)	141(36.7%)	64(16.7%)

If you get side effects during a course of antibiotics treatment you should stop taking them as soon as possible	349(90.9)	34(8.8%)	1(0.3%)

If you get some kind of skin reaction when using an antibiotic, you should not use the same antibiotic again	276(71.8%)	104(27.1%)	4(1.1%)

The unnecessarily use of antibiotics can increase the resistance of bacteria to them	301(78.4%)	27(7.1%)	56(14.5%)

Resistance to antibiotics is a worldwide problem	121(31.5%)	134(34.9%)	129(33.6%)

**Table 3:** Attitudes towards use of antibiotics among Harar city and its surrounding residents from February 1 to May 1, 2017 (n = 384).

**Statement**	**Strongly agree n(**%**)**	**Agree n(**%**)**	**Neutral n(**%**)**	**Disagree n(**%**)**	**Strongly disagree n(**%**)**
I always complete the course of treatment with antibiotics even if I feel better	310(80.7)	44(11.5)	0	18(4.7)	12(3.1)

If I feel better after a few days, I sometimes stop taking my antibiotics before completing the course of treatment	19(4.9)	90(23.5)	2(0.5)	129(33.6)	144(37.5)

I prefer to keep antibiotics at home in case there may be a need for them later	15(3.9)	33(8.6)	1(0.2)	153(39.8)	182(47.5)

It is good to be able to get antibiotics from relatives or friends without having to see a medical doctor.	17(4.4)	19(4.9)	2(0.5)	125(32.6)	221(57.6)

I prefer to be able to buy antibiotics from the pharmacy without a prescription.	18(4.7)	83(21.6)	2(0.5)	143(37.3)	138(35.9)

I prefer to use an antibiotic if I have a cough for more than a week	75(19.5)	170(44.2)	2(0.5)	78(20.3)	59(15.3)

When I have a sore throat I prefer to use an antibiotic	90(23.4)	124(32.3)	5(1.3)	147(38.3)	18(4.6)

## Data Availability

The datasets are available from the corresponding author on reasonable request.

## Additional Points

*Limitations of the Study*. This study should be interpreted cautiously for many reasons. Firstly, as we were assessing past one year experience of the subjects, bias might have been introduced. Its generalizability is also questionable since the study was conducted only in one center. In addition, since this is descriptive cross-sectional design, we were not able to identify associated factors with knowledge, attitude, and practice of the participants.
